# High prevalence of current tobacco smoking among patients with tuberculosis and people living with HIV in Jordan: A cross-sectional survey

**DOI:** 10.18332/tid/171551

**Published:** 2023-10-20

**Authors:** Ayaka Teshima, Ayah A. Shatnawi, Srinath Satyanarayana, Yousef S. Khader, Ibrahim F. Maia, Nevin C. Wilson

**Affiliations:** 1 Tobacco Control Unit, Department of Cancer Epidemiology and Prevention, Catalan Institute of Oncology (ICO), L'Hospitalet de Llobregat, Barcelona, Spain; 2 Tobacco Control Research Group, Bellvitge Biomedical Research Institute-IDIBELL, L'Hospitalet de Llobregat, Barcelona, Spain; 3 Faculty of Medicine and Health Sciences, University of Barcelona, Barcelona, Spain; 4 Migration Health Division, International Organization for Migration (IOM), Amman, Jordan; 5 Department of Community Medicine, Public Health and Family Medicine, Faculty of Medicine, Jordan University of Science and Technology, Irbid, Jordan; 6 Department of Chest Diseases and Migrant Health, Ministry of Health, Amman, Jordan

**Keywords:** tuberculosis, PLHIV, tobacco use, migration health, socioeconomic status

## Abstract

**INTRODUCTION:**

Continued smoking by patients with tuberculosis (TB) and people living with HIV (PLHIV) leads to adverse treatment outcomes. Estimates of tobacco use among the population are scarce in the Eastern Mediterranean region, where the burden of TB and HIV is also low but highly variable. This study determined the prevalence of current smoking and assessed factors associated with current smoking among patients with TB and PLHIV in Jordan.

**METHODS:**

We analyzed data from the Jordan Knowledge, Attitude, and Practices survey in 2021. Information on current tobacco use, including products and frequency of smoking, was collected from 452 patients with TB and 152 PLHIV. We performed multivariable logistic regression to assess the sociodemographic characteristics independently associated with current smoking.

**RESULTS:**

Prevalence of current smoking was 43.8% among TB patients and 67.8 % among PLHIV, and conventional cigarettes were the most used tobacco products. The prevalence of current smoking among patients with TB was higher among males (AOR=8.20; 95% CI: 5.05–13.32), Jordanians (AOR=5.37; 95% CI: 2.66–10.86) and Syrians (AOR=4.13; 95% CI: 1.60–10.67), and those experiencing financial difficulties (AOR=2.83; 95% CI: 1.69–4.74). The prevalence of current smoking among PLHIV was higher in those with financial difficulties (AOR=3.13; 95% CI: 1.19–8.27).

**CONCLUSIONS:**

Nearly half of the patients with TB and PLHIV were current tobacco smokers, higher than the general population. There is an urgent need to investigate the reasons for such a high smoking prevalence and introduce and strengthen smoking cessation services under the TB and HIV control programs.

## INTRODUCTION

Smoking, tuberculosis (TB), and human immunodeficiency virus (HIV) infection persist as three of the most critical public health challenges globally^1^. World Health Organization (WHO) estimates that more than 20% of global TB incidence in 2020 may be attributable to tobacco smoking, and it is projected to cause an additional 18 million TB cases by 2050^2,3^. Available evidence has shown that smoking aggravates TB symptoms and increases the probability of drug resistance, recurrent TB, and poor TB treatment outcomes, including death^4,5^. In addition, exposure to secondhand smoke will also significantly increase the risks of TB infection and TB disease in the families and the close contacts of patients with TB^1,4,6^.

Tobacco smoking is also hazardous for people living with HIV (PLHIV) as smokers are more likely than non-smokers to develop cardiovascular disease (CVD), malignant neoplasms, chronic obstructive pulmonary disease (COPD), and serious HIV-related infections, including bacterial pneumonia^7,8^. Smokers are also more likely to have a poorer response to HIV treatment, a greater chance of developing a life-threatening illness and a shorter lifespan than people with HIV who do not smoke^8^. Furthermore, the average years of life lost for smokers with HIV have been estimated as 12 years, which is more than twice the number of years lost by HIV infection alone^8^.

The countries of the Middle East region are experiencing a smoking epidemic^9,10^. The Eastern Mediterranean Region (EMR) is one of two WHO regions with the fastest-growing consumption of tobacco products, where the prevalence of tobacco use is expected to increase by 25% by the year 2025, compared with a decrease in Asia, North America, and Europe^11^. The prevalence of tobacco use in Jordan is 65.3% for men and 16.4% for women, considered one of the world’s most significant tobacco users in 2019^12^. The prevalence of tobacco smoking among women has increased by 4% over a decade, approaching the levels seen in Western countries^13^.

Jordan has made significant progress in improving TB and HIV treatment services, and is a low TB and HIV burden country, with a TB incidence rate of 4 per 100000 population and an HIV prevalence rate of 0.02% in the general population^14,15^. However, the burden of TB and HIV is concentrated among certain high-risk vulnerable groups, including economic migrants from high TB burden countries of Asia and Africa, and refugees from neighboring conflict-affected countries^16,17^. Previous studies have suggested a notable prevalence of tobacco use among populations with TB and HIV in countries with a high burden of these diseases, but current information on tobacco use among TB patients and PLHIV is unknown in Jordan. This study will help TB and HIV programs assess if tobacco smoking affects optimal TB and HIV treatment outcomes and integrate tobacco cessation services as part of treatment services in both programs. In this context, this study was undertaken to determine the prevalence of current tobacco smoking among patients with TB and PLHIV and assess the sociodemographic characteristics associated with current smoking among patients with TB and PLHIV in Jordan.

## METHODS

### Study design and data source

We conducted a cross-sectional study using data on tobacco use collected from part of the Knowledge, Attitude, and Practice (KAP) survey. This survey was funded by the International Organization for Migration (IOM) through a grant from the Global Fund and conducted by the Eastern Mediterranean Public Health Network (EMPHNET) research team using a semi-structured questionnaire. Convenience sampling was used to recruit participants, and the response rate was 99%. The number of patients with TB and PLHIV selected during the study period represents the vast majority of patients under treatment in 2021. The English version of the entire questionnaire is available in the Supplementary file. The KAP survey was translated to Arabic from English using the backward–forward translation method by two bilingual experts at the EMPHNET translation department. All interviews were conducted face-to-face or by telephone by researchers in an appropriate language to collect self-reported data.

For enrolling patients with TB into the KAP survey, the sample size was calculated using a single population proportion formula, assuming that the expected proportion of patients with adequate knowledge is 50%. At 95% confidence interval (CI), 5% precision, and 10% non-response rate, the minimum sample size was calculated at 428 patients. The KAP survey for patients with TB enrolled all unique patients with TB (N=452) who attended any of the nine TB health centers (covering all TB treatment facilities) in four governorates, namely Amman, Mafraq, Irbid, and Zarqa during the months of June to September 2021. Although no sample size was estimated for enrolling PLHIV in the KAP survey, the KAP survey enrolled all unique PLHIV (N=152) who visited voluntary counselling and testing (VCT) from all governorates in Jordan for follow-up and anti-retroviral treatment during April and September 2021.

### National program for TB and HIV control

The National Tuberculosis Program (NTP) in Jordan adopted the WHO Directly Observed Treatment, Short-course (DOTS) strategy in the mid-nineties. At the central level, the national program unit located in Amman is integrated with the foreign health division. It is responsible for developing TB care policies and their country-wide implementation. The governorate or second level comprises centers in the respective 12 governorates responsible for providing TB prevention, care, and control services as defined in the national policy. A national TB reference laboratory supports testing for drug resistance (first and second line) and coordinates quality control for the network of labs providing TB diagnosis in the country. A third level includes health centers in the periphery where a trained nurse supports treatment monitoring and patient support, as well as referring new cases to the program for an assessment and a diagnosis. While national policy requires non-nationals without a refugee status to be deported on diagnosis of TB, the NTP continues to diagnose and treat a significant number of non-nationals with TB. TB treatment is free of charge^18^.

The National AIDS Program (NAP) was established under the Ministry of Health after the first case of AIDS was recorded in Jordan in 1986. The National AIDS Committee (NAC) was formed in response and continued working for some period. The NAC was the primary group formulating national HIV/AIDS policy, with broad participation from the public, private, academic, and non-governmental sectors (NGOs). At the sub-national level, there are currently 12 Focal Points for HIV/AIDS in the central governorates of the country (12 governorates). The National AIDS Strategy 2005–2009 was established to focus on the adolescent population to acquire life skills, participate in voluntary counselling and testing, and free antiretroviral medicines. The National Strategic Plan (NSP) on HIV and AIDS (2012–2016) was the second national framework that directed the response. No strategic framework governing the national reaction between 2016 and 2021, when a revised framework was developed and currently guides the national response^19^. Treatment and care for people living with HIV have been provided free of charge by the government since 2003. Amman’s Care and Treatment Centre is the leading service point for treatment care and support (TCS) through Governorate Health Directorates. Antiretroviral therapy (ART) is covered by public health insurance, including for specific groups of non-Jordanian spouses of Jordanian citizens.

### Measures

The KAP survey assessed current tobacco smoking status by the following questions: ‘Do you now smoke cigarettes?’ and ‘do you now smoke tobacco in a waterpipe?’. The possible options were: ‘every day’, ‘some days’, and ‘not at all’. Current tobacco smokers were defined as respondents who answered ‘every day’ or ‘some days’ in smoking cigarette tobacco and/or waterpipes. We considered four categories of smokers: current tobacco smokers (cigarettes and/or waterpipes), current cigarette-only smokers, current waterpipe-only smokers, and current dual smokers.

The survey collected self-reported demographic data on age (<18; 18–35; 36–55; >55 years), sex (male, female), Nationality (Jordanian, Syrian, Palestinian, Other), and education level low (no formal education, primary, middle school), moderate (high school and secondary vocational) and high (university and postgraduate), marital status (single, separated, married), and employment status (employed, unemployed). Also, financial difficulty was assessed based on whether or not they had to borrow money to pay bills on time in the last month (yes, no) as a proxy for socioeconomic status.

The TB treatment-related information collected included a number of follow-up TB center visits in the past (1, 2–5, >5 times) and TB type (pulmonary TB, drug-resistant TB, extrapulmonary TB). Participants were asked about the TB treatment stage. Respondents could select up to four options: ‘Never had TB treatment’, ‘Currently on TB treatment’, ‘Completed TB treatment within the last year’, and ‘Completed TB treatment over one year ago’. The HIV information collected included data on the HIV stage and whether the participants have experienced HIV-related symptoms (no symptoms, have symptoms). Family HIV status was assessed by the following question: ‘Do any of your family members have HIV/AIDS?’ (yes, no). Participants were also asked whether they have any chronic diseases other than HIV (yes, no).

### Statistical analysis

We first calculated the prevalence of current smoking (%) with 95% Confidence Interval (95% CI) and disaggregated by cigarette-only, waterpipe-only, and dual use. We calculated the relative prevalence of current smoking (%) in various sociodemographic sub-groups. A bivariate and multivariable logistic regression model was performed to determine factors associated with current tobacco smokers (conventional cigarettes and/or waterpipe) and presented as odds ratio (OR) and adjusted OR (AOR), with 95% CI, respectively. The covariates in the multivariable logistic regression included those statistically associated at the bivariate level and those that we presumed theoretically were likely associated with tobacco smoking in the country. Accordingly, the variables in the multivariable logistic regression model for patients with TB included sex, age, education level, marital status, occupation, financial difficulties, number of follow-up TB center visits, type of TB, and stage of TB treatment. The variables in the multivariable logistic regression model for HIV patients included sex, age, education level, marital status, employment status, financial difficulties, chronic disease status, family’s HIV status, and HIV symptoms. We also used a Bayesian information criterion and an Akaike information criterion to decide the most appropriate model. We confirmed no multicollinearity in the regression analyses by checking all covariates’ variance inflationary factor (VIF). There were no missing data because of the rigorous procedures by researchers from the EMPHNET team. In the analysis, all tests of statistical significance were two-sided, and p<0.05 was considered statistically significant. All statistical analyses were conducted in R version 4.1.3.

## RESULTS

### Study population

The sociodemographic and clinical characteristics of 452 patients with TB and 152 PLHIV enrolled in the KAP study are presented in Table 1. The mean age of patients with TB was 37 (SD=13.2) years, and that of PLHIV was 41.4 (SD=11.2) years. About 44% of the patients with TB and 81% of PLHIV were males. Regarding nationality, 60% of patients with TB and 93% of PLHIV were Jordanians, and 44% of the patients with TB and 55% of the HIV patients had a high education level. A large proportion of TB and HIV patients had financial difficulties, 46% and 61%, respectively. The main type of TB was pulmonary TB (63%). Almost 70% of patients with TB were currently on treatment. Nearly 78% did not have any HIV-related symptoms (78%), chronic disease (84%), or family with HIV (87%). To confirm differences, Supplementary file Table 1 and Supplementary file Table 2 illustrate the demographic characteristics of patients with TB before and during the COVID-19 pandemic by comparing the Middle East Response (MER) data which covered all patients with TB by IOM and the KAP survey. Although age distribution and the total number of individuals were almost the same during the duration of the pandemic, the proportion of migration significantly decreased from 61 % in 2019 to 28% in 2021.

**Table 1:** Sample characteristics of patients with TB and people living with HIV (PLHIV) enrolled in the KAP survey, Jordan, 2021

*Variables*	*Patients with TB (N=452)*		*Variables*	*PLHIV (N=152)*	
	*n*	*%*		*n*	*%*
**Sex**			**Sex**		
Female	251	56	Female	29	19
Male	201	44	Male	123	81
**Age** (years)			**Age** (years)		
<18	18	4			
18–35	215	48	18–35	51	34
36–55	171	38	36–55	85	56
>55	48	11	>55	16	11
**Migration status**			**Migration status**		
Jordanian	271	60	Jordanian	10	7
Other	116	26	Non-Jordanian	142	93
Syrian	54	12			
Palestinian	11	2			
**Education level**			**Education level**		
Low	173	38	Low	21	14
Moderate	197	44	Moderate	47	31
High	82	18	High	84	55
**Marital status**			**Marital status**		
Married	303	67	Married	68	45
Single	149	33	Single	57	38
			Separated	27	18
**Financial difficulty**			**Financial difficulty**		
No	244	54	No	59	39
Yes	208	46	Yes	93	61
**Employment status**			**Employment status**		
Unemployed	238	53	Unemployed	63	41
Employed	214	47	Employed	89	59
**TB treatment stage**			**HIV stage**		
First time	14	3	No symptoms	119	78
Currently on treatment	334	74	Have symptoms	23	15
Completed within a year	45	10			
Completed over a year ago	59	13			
**TB type**			**Chronic diseases**		
Extrapulmonary TB	163	36	No	128	84
Pulmonary TB	283	63	Yes	24	16
**Number of follow-up TB center visits**			**Family HIV status**		
1	147	33	No	132	87
2–5	137	30	Yes	20	13
≥5	168	37			

### Prevalence and frequency of current tobacco use

Table 2 shows the prevalence and frequency of current tobacco smoking according to tobacco products among patients with TB and PLHIV. The overall prevalence of current smoking was 43.8% (95% CI: 39.2–48.5) in patients with TB and 67.8% (95% CI: 59.6–75.0) among PLHIV. Men (31.4%; 95% CI: 27.2–35.9) were more likely to be current smokers than women (12.3%; 95% CI: 9.6–15.9) among patients with TB. Similarly, among PLHIV, 61.8% (95% CI: 53.6–69.5) were male smokers and 5.9% (95% CI: 2.9–11.3) were female smokers. The most common tobacco product smoked was cigarette-only at 36.3% (95% CI: 31.9–40.9) for patients with TB and 55.9 % (95% CI: 47.7–63.9) for PLHIV. Most cigarette smokers used these products daily, in patients with TB and PLHIV. However, among current waterpipe-smokers they were used occasionally. Differences in tobacco smoking prevalence and frequency according to nationalities among patients with TB are given in Supplementary file Table 3.

**Table 2:** Tobacco smoking prevalence and frequency among patients with TB and PLHIV in Jordan, 2021

	*Patients with TB (N=452)*		*PLHIV (N=152)*		
	*n (%)*	*95% CI*	*n (%)*	*95% CI*	*p^a^*
**Current tobacco use**					
Overall	198 (43.8)	39.2–48.5	103 (67.8)	59.6–75.0	<0.001
**Current cigarette-only use**					
Overall	164 (36.3)	31.9–40.9	85 (55.9)	47.7–63.9	<0.001
Daily use	115 (25.4)	21.5–29.8	64 (42.1)	34.2–50.4	<0.001
Occasional use	49 (10.8)	8.2–14.2	21 (13.8)	8.9–20.6	0.398
**Current waterpipe-only use**					
Overall	71 (15.7)	12.5–19.5	36 (23.7)	17.3–31.4	0.035
Daily use	9 (2.0)	1.0–3.9	8 (5.3)	2.5–10.5	0.068
Occasional use	62 (13.7)	10.8–17.3	28 (18.4)	12.8–25.7	0.201
**Current dual use**					
Overall	37 (8.2)	5.9–11.2	18 (11.8)	7.4–18.3	0.233
Daily use	4 (0.9)	0.3–2.4	3 (2.0)	0.5–6.1	0.517
Occasional use	33 (7.3)	5.2–10.2	15 (9.9)	5.8–16.0	0.401

a Chi-squared test.

**Figure 1:**
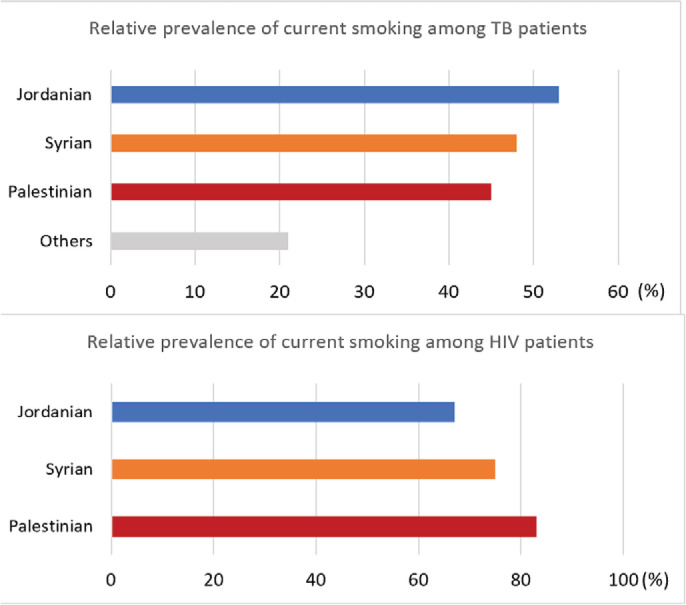
The relative prevalence of current tobacco use by migration status among patients with TB (N=452) and PLHIV (N=152) in Jordan, 2021

The relative prevalence of current tobacco use among patients with TB and PLHIV, disaggregated by nationality, is given in Figure 1. Among patients with TB, prevalence of current tobacco was highest in Jordanians (53%, 143/271), followed by Syrians (48%, 26/54) and Palestinians (45%, 5/11). Among PLHIV, Palestinians (83%, 5/6) had the highest proportion of current smokers, followed by Syrians (75%, 3/4) and Jordanians (67%, 95/142).

### Factors associated with the current tobacco use

**Figure 2:**
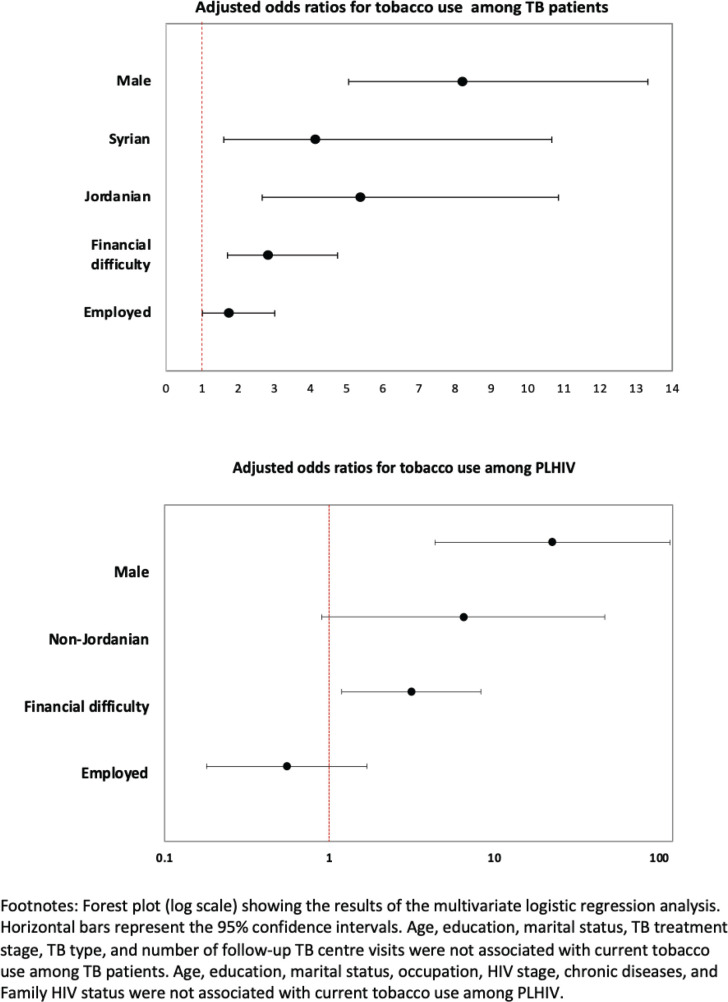
Forest chart showing the association of factors with current tobacco use among TB patients (N=452) and PLHIV (N=152) in Jordan, 2021

The sociodemographic characteristics associated with current tobacco smoking among patients with TB and PLHIV are given in Tables 3 and 4. Among patients with TB, men were more likely than women to be current smokers (AOR=8.20; 95% CI: 5.05–13.32). Similarly, Jordanians (AOR=5.37; 95% CI: 2.66–10.86) and Syrians (AOR=4.13; 95% CI: 1.60–10.67) were more likely to smoke than migrants from other countries. Patients with TB with financial difficulties had 2.83 times greater odds of current tobacco use than those who reported having no difficulty paying bills (AOR=2.83; 95% CI: 1.69–4.74). Apart from these, there was no clear association between current smoking prevalence and TB-related variables such as TB treatment stage, TB type, and number of follow-up visits to TB centers. Among PLHIV, the characteristics associated with higher odds of current tobacco smoking include male gender (AOR=22.46; 95% CI: 4.36–115.65) and those having financial difficulties (AOR=3.13; 95% CI: 1.19–8.27) (Figure 2).

**Table 3:** Characteristics of registered patients with TB and factors associated with current smoking in Jordan, 2021

*Variables*	*All*	*Current smokers*		*OR (95%CI)*	*AOR (95% CI)*	*p*
	*n*	*n*	*%*			
**Total**	452	198	44			
**Sex**						
Female (Ref.)	251	56	22	1	1	
Male	201	142	71	8.38 (5.48–12.82)	8.20 (5.05–13.32)	0.000
**Age** (years)						
<18 (Ref.)	18	4	22	1	1	
18–35	215	89	41	2.47 (0.79–7.76)	4.62 (1.21–17.73)	0.026
36–55	171	84	49	3.38 (1.07–10.68)	5.25 (1.31–21.11)	0.020
>55	48	21	44	2.72 (0.78–9.49)	3.31 (0.74–14.72)	0.116
**Migration status**						
Other (Ref.)	116	24	21	1	1	
Syrian	54	26	48	3.56 (1.77–7.15)	4.13 (1.60–10.67)	0.003
Palestinian	11	5	45	3.19 (0.90–11.36)	4.31 (0.90–20.67)	0.068
Jordanian	271	143	53	4.28 (2.58–7.12)	5.37 (2.66–10.86)	<0.001
**Education level**						
Low (Ref.)	173	72	42	1	1	
Moderate	197	91	46	1.20 (0.80–1.82)	1.22 (0.72–2.08)	0.459
High	82	35	43	1.04 (0.61–1.78)	0.89 (0.44–1.78)	0.741
**Marital status**						
Married (Ref.)	303	131	43	1	1	
Single	149	67	45	1.07 (0.72–1.59)	1.41 (0.80–2.50)	0.235
**Financial difficulty**						
No (Ref.)	244	81	33	1	1	
Yes	208	117	56	2.59 (1.77–3.79)	2.83 (1.69–4.74)	<0.001
**Employment status**						
Unemployed (Ref.)	238	98	41	1	1	
Employed	214	100	47	1.25 (0.86–1.82)	1.74 (1.01–3.00)	0.045
**TB treatment stage**						
First time (Ref.)	14	4	29	1	1	
Currently on treatment	334	146	44	0.52 (0.16–1.68)	0.65 (0.13–3.31)	0.604
Completed within a year	45	20	44	1.03 (0.55–1.93)	0.83 (0.36–1.91)	0.670
Completed over a year ago	59	28	47	1.16 (0.67–2.03)	1.37 (0.65–2.91)	0.407
**TB type**						
Extrapulmonary TB (Ref.)	163	68	42	1	1	
Pulmonary TB	283	127	45	1.14 (0.77–1.68)	0.84 (0.50–1.39)	0.494
**Number of follow-up TB center visits**						
1 (Ref.)	147	56	38	1	1	
2–5	137	65	47	1.47 (0.91–2.35)	0.99 (0.54–1.85)	0.987
>5	168	77	46	1.37 (0.88–2.16)	0.86 (0.45–1.66)	0.661

AOR: adjusted odds ratio; mutually adjusted for all covariates included in the table.

**Table 4:** Characteristics of registered PLHIV and factors associated with current smoking in Jordan, 2021

*Variables*	*All*	*Current smokers*		*OR (95% CI)*	*AOR (95% CI)*	*p*
	*n*	*n*	*%*			
**Total**	152	103	68			
**Sex**						
Female (Ref.)	29	9	31	1	1	
Male	123	94	76	7.20 (2.96–17.54)	22.46 (4.36–115.65)	<0.001
**Age** (years)						
18–35 (Ref.)	51	35	69	1	1	
36–55	85	57	67	0.93 (0.44–1.96)	1.09 (0.39–3.07)	0.869
>55	16	11	69	1.01 (0.30–3.38)	0.69 (0.13–3.50)	0.650
**Migration status**						
Jordanians (Ref.)	142	95	67	1	1	
Non–Jordanians	10	8	80	1.98 (0.40–9.69)	6.49 (0.90–46.64)	0.063
**Education level**						
Low (Ref.)	21	14	67	1	1	
Moderate	47	30	64	0.88 (0.30–2.61)	0.93 (0.24–3.60)	0.919
High	84	59	70	1.18 (0.43–3.27)	1.64 (0.40–6.75)	0.495
**Marital status**						
Married (Ref.)	68	43	75	1	1	
Separated	27	17	63	0.99 (0.39–2.49)	2.98 (0.74–12.03)	0.126
Single	57	43	63	1.79 (0.82–3.89)	1.75 (0.61–4.99)	0.295
**Financial difficulty**						
No (Ref.)	59	35	59	1	1	
Yes	93	68	73	1.87 (0.93–3.73)	3.13 (1.19–8.27)	0.021
**Employment status**						
Unemployed (Ref.)	63	37	59	1	1	
Employed	89	66	74	2.02 (1.01–4.02)	0.55 (0.18–1.69)	0.300
**HIV Stage**						
No symptoms (Ref.)	119	81	68	1	1	
Have symptoms	23	15	65	0.88 (0.34–2.25)	1.43 (0.47–4.41)	0.531
**Chronic diseases**						
No (Ref.)	128	86	67	1	1	
Yes	24	17	71	1.19 (0.46–3.08)	0.83 (0.24–2.81)	0.759
**Family HIV status**						
No (Ref.)	132	91	69	1	1	
Yes	20	12	60	0.68 (0.26–1.78)	1.76 (0.39–7.94)	0.459

AOR: adjusted odds ratio; mutually adjusted for all covariates included in the table.

## DISCUSSION

This is the first study to determine the prevalence of current smoking and factors associated with it among patients with TB and PLHIV, in Jordan. Our analysis found that 43.8% of patients with TB and 67.8% of PLHIV were current smokers, higher than the general population (41.0%) according to the nationally representative Jordan National Stepwise Survey (STEPs) conducted in 2019^12^. The likelihood of current tobacco smoking among patients with TB was higher among males, and those aged ≥18 years compared with those <18 years, Jordanians and Syrians compared to people from other nationalities, those experiencing financial difficulties, and those who were employed than unemployed. Males and those with financial difficulties among PLHIV were more likely to smoke.

High smoking prevalence from our results is consistent with previous studies conducted in other high TB and HIV burden countries, such as India, China, Bangladesh, Pakistan, and Sub-Saharan Africa^8,20-23^, which have found higher smoking prevalence among patients with TB and PLHIV.

The study has the following implications for policy and practice in the Jordanian context.

First, the reasons for high levels of smoking prevalence are unknown and need to be investigated to ascertain whether high levels of smoking are a cause of TB disease or a consequence of TB and HIV diagnosis^7,24^. Evidence from high-income countries (HICs) has consistently shown that individuals with low socio-economic status (SES) have higher rates of smoking and lower rates of quitting compared to those with high SES^25^. In contrast, among general populations in the Eastern Mediterranean region, a gradient in smoking rates by education has been observed, but there is an absent association with wealth, and in some cases, the wealthiest individuals exhibit higher behavioral risk^26,27^. Our findings indicate a positive association between financial difficulties and tobacco use among both TB patients and PLHIV, which is consistent with the evidence observed in HICs^25^; however, no clear association with education level in our results is in contrast to previous surveys in the Eastern Mediterranean region^26-28^. Additionally, our results are incompatible with current literature on TB patients and PLHIV^29-31^, which have suggested that lower education, detrimental alcohol, and drug use are predictors of tobacco use, but findings on wealth are inconclusive. Alcohol and drug use behavior is also relatively rare in the region for religious reasons; therefore, TB patients and PLHIV who smoke in the region may have a distinct sociodemographic profile to cohorts in other areas and general populations.

While it is well known that smokers have higher chances of developing TB disease^4,6^, information gathered during the KAP survey indicated that patients with TB and PLHIV face high levels of stigma and discrimination after the diagnosis of these diseases. Direct associations between stigma and smoking behavior have not been extensively studied. However, existing studies have indicated that stigma is associated with substance use, social isolation, and anxiety^32,33^. These predictors have demonstrated a strong association with smoking behaviour^34^ and may partially explain one possible reason for the elevated prevalence of smoking. Patients with TB and PLHIV might be compelled to smoke excessively or take up smoking to overcome the social, economic, and mental stress induced by the diagnosis of these diseases. Further investigation will help in understanding the causes of high levels of current tobacco use and support the design of context-specific interventions to address current tobacco smoking.

Second, irrespective of the causes of high levels of current tobacco smoking, there is a need for the introduction or integration of tobacco cessation services into the TB and HIV treatment services. Tobacco cessation not only benefits patients with TB and PLHIV but also benefits the family and other close contacts by reducing exposure to secondhand smoke. In Jordan, currently, tobacco cessation services that include provision of nicotine replacement therapy (NRT) are implemented as a part of the cancer care^35^, and a similar approach needs to be adopted while introducing it as part of TB and HIV treatment services^35,36^.

Third, while including the tobacco cessation services as a part of the TB and HIV treatment services, care must be taken to ensure that the services are tailored to the sociodemographic characteristics of the patients with TB and PLHIV. Though males were more likely than females to be current tobacco smokers, about 21% of the female patients with TB and 33% of the female PLHIV were current smokers. Therefore, the cessation services need to be sensitive to the needs of not only male patients with TB and PLHIV, but also female patients with TB and PLHIV. Similarly, a significant proportion of the patients with TB an PLHIV who belong to a nationality other than Jordanian are also current tobacco smokers and therefore, care must be taken to customize the cessation services to suit persons of other nationalities. The study results also show that a significant proportion of patients with TB and PLHIV are experiencing financial difficulties in paying off their daily bills, and current tobacco use was higher among such individuals. Whether it is essential to link patients with TB to any other existing social protection schemes to reduce the tobacco smoking behavior needs further exploration.

Fourth, tobacco smoking in the general population of Jordan and other neighboring countries is increasing^10,11,28^. Tobacco smoking is one of the risk factors for TB disease, and therefore growing tobacco prevalence can negatively affect the TB burden in these countries^2^. This can hamper the achievement of the TB elimination efforts in the country and region. Therefore, the TB control program has a role in advocating with the policymakers on the need for large-scale interventions for tobacco control, which, if not done, may increase the demand for resources to provide TB services.

Finally, there is a need for establishing mechanisms for tracking the trends in tobacco cessation among patients with TB and PLHIV and to assess the effectiveness of the measures being undertaken to reduce the tobacco smoking burden in these persons. This can be done by revising the recording and reporting formats to document the tobacco consumption status during TB and HIV treatment.

### Strengths and limitations

The major strength of this study is that all TB and HIV patients attending the health facilities during the study period were enrolled with 100% participation, thereby minimizing the chances of selection bias and enhancing the reliability and generalizability of the study findings to all patients with TB and PLHIV. The study has some limitations. First, it is a cross-sectional study, and any causal relationship between predictors and smoking prevalence is difficult to establish. Second, there could be under-reporting of tobacco use, especially among women and younger persons, due to smoking measures based on self-report. However, no under-reporting trends were observed in our findings from previous studies, with estimated tobacco use ranging from 15% to 30% in adolescents and 16.4% in women. Third, the present study is based on convenience sampling, but the study is the only study that has assessed smoking among patients with TB and PLHIV and provided comprehensive information on several relevant factors in the region. Fourth, we only assessed current tobacco use regarding conventional cigarettes and waterpipes since they are the most common tobacco products used in middle-eastern countries. However, other tobacco use types might also be present due to the widespread use of e-cigarettes and heated tobacco products in recent years. Fifth, we have done this study in a setting where the prevalence of TB among HIV patients is very low and vice versa. Hence studying an association between smoking and TB/HIV co-infection in this setting with this population was not possible. Lastly, we did not quantify the length and intensity of smoking, which prevents us from assessing a dose–response relationship or the intensity of the services needed to ensure tobacco cessation.

## CONCLUSIONS

Our study highlights a substantial proportion of tobacco use among patients with TB and PLHIV with a high burden of disease from smoking. We call upon the National TB and HIV programs in Jordan to investigate the reasons for high consumption and integrate smoking cessation interventions into TB and HIV treatment services.

## Data Availability

The data supporting this research are available from the authors on reasonable request.
