# Supplementary material for: High prevalence of current tobacco smoking among patients with tuberculosis and people living with HIV in Jordan: A cross-sectional survey
Source: Tob Induc Dis. 2023 Oct 20;21:136. doi: 10.18332/tid/171551 (PMC10587893; doi:10.18332/tid/171551)
Supplement: Supplementary file 1 [file TID-21-136-s1.pdf]

Supplementary

Supplementary Table 1. Comparison of the Middle East Response (MER) data and the Knowledge, Attitude and Practices (KAP) survey demographic characteristics of patients with TB before (2019) and during COVID-19 (2020 and 2021)

Year of survey	MER 2019 [a]		MER 2020 [a]			KAP 2021 [b]	
Total	454 [c]		250 [c]			452	
	n (%)	*95%CI*	*n (%)*	*95%CI*	*p* [d]	*n (%)*	*95%CI*
**Age**					0.428		
0-14 years	36 (8)	5.7-10.9	15 (6)	3.5-9.9		5 (1)	0.4−2.7
> 14 years	418 (92)	89.1-94.3	235 (94)	90.1-96.5		447 (99)	97.3-99.6
**Sex**					**0.009**		
Male	136 (30)	25.8-34.4	100 (40)	33.9-46.4		201 (44)	39.8-49.2
Female	318 (70)	65.6-74.2	150 (60)	53.6-66.1		251 (56)	50.8-60.2
**Migration Status**					0.429		
Jordanian	179 (39)	34.9-44.1	107 (43)	36.6-49.2		271 (60)	55.3-64.5
Non-Jordanian	275 (61)	55.9-65.1	143 (57)	50.8-63.4		181 (40)	35.5-44.7

Footnote:

a: Middle East Response (MER) data integrates information necessary for the elimination of the three major infectious diseases such as TB, HIV and Malaria in the Middle East region through the intervention of the International Organization for Migration (IOM).

b: Knowledge, Attitude and Practices survey.

c: Number of notified cases of all forms of TB (i.e., bacteriologically confirmed + clinically diagnosed) including new and relapse cases. In the MER data in 2020, due to the COVID 19 pandemic and the curfew imposed by the government from March until June. The National Tuberculosis Programme (NTP) and Chest Diseases Centres were closed completely at the beginning of the curfew and some of the NTP staff were deployed to the COVID activities in addition to the suspension of new labour migrants recruiting which have resulted in decreasing the number of TB notified cases reported.

d: Chi-squared test.

Supplementary Table 2. Comparison of current smoking prevalence by nationality in the nationally representative Jordan National Stepwise Survey (STEPs) in 2019 and KAP surveys in 2021

Survey type	STEPs survey		KAP survey		KAP survey		
Target population	General population [a]		TB patients [b]		PLHIV [b]		
	N	*n (%)*	*N*	*n (%)*	*N*	*n (%)*	*p* [c]
Overall	5713	2342 (41.0)	452	198 (43.8)	152	103 (67.8)	**0.011**
Jordanian only	2910	1222 (42.0)	271	143 (52.8)	142	95 (66.9)	**0.008**
Syrian only	2803	902 (32.2)	54	26 (48.1)	4	3 (75)	0.066

Footnote:

a: Smoking status among general population were collected from the Jordan National Stepwise Survey (STEPs) survey 2019. The STEPs survey population included Jordanian and Syrian

adults of both genders, aged 18 to 69 years old, residing in Jordan. The Jordanian sample was captured from all the 12 governorates of Jordan to enhance the geographical representation of the sample. Whereas the Syrian refugee's sample included those residing in four governorates, and only those residing outside refugee camps. Tobacco smoke includes manufactured cigarettes, hand-rolled cigarettes, shisha, pipe, and cigars. There is no overlap between prevalence of tobacco smokers and e-cigarettes/vaping products users. These are two independent groups, which are only counted once. Current refers to daily and less than daily.

b: Smoking status among TB and PLHIV population were collected from the Knowledge, Attitude and Practices (KAP) survey 2021.

c: One-way ANOVA Test

Supplementary file Table 3. Differences in tobacco smoking prevalence and frequency according to nationalities among patients with TB (N=452), in Jordan, 2021 from KAP survey

	Entire sample		Jordanian		Syrian		Palestinian		Others	
**Total**	N=452		N=271		N=54		N=11		N=116	
	n, % (95 % CI)									
**Current tobacco smoking**										
Prevalence	198	43.8(39.2 - 48.5)	143	31.6(27.4 - 36.2)	26	5.8(9.2 - 15.4)	5	1.1(1.3 - 4.4)	24	5.3(3.5 - 7.9)
**Current cigarette-only smoking**										
Prevalence	164	36.3(31.9 - 40.9)	118	26.1(22.2 - 30.5)	23	5.1(3.3 - 7.6)	5	1.1(0.4 - 2.7)	18	4.0(2.4 - 6.3)
Daily use	115	25.4(21.5 - 29.8)	87	19.2(15.8 - 23.3)	13	2.9(1.6 - 5.0)	4	0.9(0.3 - 2.4)	11	2.4(1.3 - 4.4)
Non-Daily use	49	10.8(8.2 - 14.2)	31	6.9(4.8 - 9.7)	10	2.2(1.1 - 4.2)	1	0.2(0.0 - 1.4)	7	1.5(0.7 - 3.3)
**Current waterpipe-only smoking**										
Prevalence	71	15.7(12.5 - 19.5)	55	12.2(9.4 - 15.6)	4	0.9(0.3 - 2.4)	2	0.4(0.1 - 1.8)	10	2.2(1.1 - 4.2)
Daily use	9	2.0(1.0 - 3.9)	7	1.5(0.7 - 3.3)	0	0.0(0.0 - 0.0)	1	0.2(0.0 - 1.4)	1	0.2(0.0 - 1.4)
Non-Daily use	62	13.7(10.8 - 17.3)	48	10.6(8.0 - 13.9)	4	0.9(0.3 - 2.4)	1	0.2(0.0 - 1.4)	9	2.0(1.0 - 3.9)
**Current dual smoking**										
Prevalence	37	8.2(5.9 - 11.2)	18	6.6(4.6 - 9.4)	1	0.2(0.0 - 1.4)	2	0.4(0.1 - 1.8)	10	0.9(0.3 - 2.4)
Daily use	4	0.9(0.3 - 2.4)	1	0.4(0.1 - 1.8)	0	0.0(0.0 - 0.0)	1	0.2(0.0 - 1.4)	1	0.2(0.0 - 1.4)
Non-Daily use	33	7.3(5.2 - 10.2)	17	6.2(4.2 - 8.9)	1	0.2(0.0 - 1.4)	1	0.2(0.0 - 1.4)	8	0.7(0.2 - 2.1)

Appendices

Questionnaires. TB- related KAP questionnaire among TB patients

Interview Date (day/mon/yr): ___ ___/____ ___/2021
Interview Time: ___________________________ am pm
Interviewer Name:________________________________
Center/clinic name: _______________________ Location of the center (City) _____________

A. Patients' socio-demographic Characteristics

1. Gender: 1. Male 2. Female	2. What is your age? [ ] year
3. What is your marital status?<br>1. Single 2. Engaged 3. Married 4. Separated 5. Divorced 6. Widow	
4. What is the highest education level you have obtained?<br>1. 1. No formal education<br>2. 2. Completed primary school<br>3. 3. Completed middle school<br>4. 4. Completed high school<br>5. 5. Completed 1-2-year post-secondary/vocational<br>6. 6. Completed university/college<br>7. 7. Completed postgraduate degree	
5. What is your nationality?<br>1. 1. Jordanian 2. Syrian (urban) 3. Syrian (Camp) 4. Palestinian 4. Others, __________	
6. Which of the following best describe your employment status?<br>1. Full-time employed in the workforce (including self-employed)<br>2. Part-time employed in the workforce (including self-employed)<br>3. Unemployed<br>4. Retired or on a pension<br>5. Full-time student<br>6. Part-time student<br>7. Housewife/ Home duties	
7. What is the best estimate of your monthly household income? [ ] JD<br>*We ask this for statistical purposes. We assure your responses will remain strictly confidential.*	
8. In the last month, did you have to borrow money to pay any important bills on time, such as electricity, telephone or rent bills?<br>1. 1. Yes 2. No 3. Don't know	
9. Residency/Governorate: 1. Amman 2. Mafraq 3. Zarqa 4. Irbid 5. Others_______	
10. Do you live with your family? 1. I live alone 2. I live with my family	
11. Do anyone of your family members have been diagnosed with TB? 1. Yes 2. No 3. I don't know	
12. Do you now smoke cigarettes? 1. Every day 2. Some days 3. Not at all	

13. Do you now smoke tobacco in a waterpipe? 1. Every day 2. Some days 3. Not at all

14. Which of the following statements is accurate about you?
    1. Completed TB treatment over one year ago
    2. Completed TB treatment within the last year
    3. Currently on TB treatment
    4. Never had TB treatment
15. How many times you attended the TB clinic in the last year?
    1. This is first time
    2. Two to five times
    3. More than five times
16. What type of TB were you last diagnosed with?
    1. Pulmonary TB
    2. Multidrug-Resistant TB
    3. Extensively Drug-Resistant TB
    4. Extrapulmonary TB
    5. Don't Know

B. Patients' knowledge of TB

B1. What is the main source of your information about tuberculosis? (select one)
1. Radio or TV
2. Newspapers or magazines
3. Healthcare provider/ Health care facility
4. Family, friends, neighbors, or colleagues
5. Teachers
6. Internet

B2. What is the cause of TB infection? 1. Fungal 2. Viral 3. Bacterial 4. I don't know

B3. Is TB highly infectious disease? 1. Yes 2. No 3. I don't know

B4. Is TB a curable disease? 1. Yes 2. No 3. I don't know

B5. Is TB preventable? 1. Yes 2. No 3. I don't know

B6. Which one of the followings are signs and symptoms of TB?

	1. Yes	2. No	3. I don't know
1. Cough that lasts longer than 2 weeks			
2. Coughing up blood			
3. Weight loss			
4. Fever without clear cause that lasts for more than 7 days			
5. Ongoing fatigue			
6. Severe headache			
7. Night sweating			
8. Shortness of breath			
9. Chest pain			
10. Nausea			
11. loss of appetite			

B7. How TB is transmitted from an infected person to others?

	1. Yes	2. No	3. I don't know
1. Through the air when a person with TB coughs or sneezes			
2. Sharing the patient, a poorly ventilated room or place			
3. Through handshakes			
4. By sharing dishes, plates, cups, and spoons or eating from the same plate			
5. Through touching items in public places (doorknobs, handles in transportation, etc.)			
6. Through smoking cigarettes			

B8. Mainly, how Tuberculosis is diagnosed?

	1. Yes	2. No	3. I don't know
1. Sputum examination			
2. Chest X-ray			
3. Blood examination			
4. Stool & Urine examination			

B9. How can a person prevent getting TB?

	1. Yes	2. No	3. I don't know
1. Covering mouth and nose when being in crowded places			
2. Avoid sharing dishes and personal items			
3. Through good nutrition			
4. Washing hands after touching items in public places			
5. Closing windows at home			
6. Safe disposal of sputum			
7. Ventilation of living room			
8. Vaccination of children			

B10. Which body parts could be affected with TB?

	1. Yes	2. No	3. I don't know
1. Lung			
2. Intestine			
3. Bone			
4. Lymph nodes			
5. Others			

B11. One should be examined if a prolonged cough with sputum persists for more than 2 weeks.

1. Yes 2. No 3. Don't know

B12. What is the duration of TB treatment?

1. 12 months
2. 8 months
3. 6 months
4. 4 months

B13. What are the consequences of interrupted treatment?

	1. Yes	2. No	3. I don't know
1. The patient will not be cured			
2. Death			
3. Drug resistance			
4. No effect			

B14. What is the major side effect of TB drugs?

	1. Yes	2. No	3. I don't know
1. Vomiting			
2. Dizziness			
3. Jaundice			
4. Itching of skin			

B15. When will you stop taking TB drugs?

	1. Yes	2. No	3. I don't know
1. Once TB symptoms are disappeared			
2. When feeling healthy			
3. After declaring TB cured by health worker			

B16. In your opinion, how serious is TB disease?

1. Very serious
2. Somewhat serious
3. Not very serious

C. Knowledge and attitude of Multi-drug Resistance (MDR-TB)

C1. Did you hear about Multi-drug Resistance (MDR-TB)? 1. Yes 2. No

If you heard about MDR-TB, please answer the questions below. Otherwise, skip to questions in section D.

C2. Where have you heard about MDR-TB?

	1. Yes	2. No	3. I don't know
1. From health workers			
2. From mass media (television, radio, and taps)			
3. From printed materials			
4. From friends, family, and school			
5. Internet			

C3. What is the meaning of multidrug resistance tuberculosis?

	1. Yes	2. No	3. I don't know
1. Tuberculosis disease caused by a strain of TB that is resistant to at least two anti-TB drugs			
2. Tuberculosis disease that is resistant to one anti-TB drug			
3. Form of TB that requires treatment with expensive drugs			
4. Form of TB that requires treatment which gives more side effects			
5. Resistance due to default of anti TB or not completing anti-TB drugs.			

C4. What are the possible consequences of not completing TB treatment or poor adherence to TB medications?

	1. Yes	2. No	3. I don't know
1. The TB is not cured, and it will come back again			
2. The patient may die			
3. Patient may develop multidrug-resistant TB (MDR-TB)			
4. Others			

C5. What is the duration of treatment of MDR-TB?

1. Don't know
2. 6 - 8 months
3. 10-12 months
4. 18-24 months

C6. Who is at high risk to develop MDR-TB?

	1. Yes	2. No	3. I don't know
1. TB patient with retreatment regimen failure			
2. Patients with close contact history with a known MDR-TB			
3. TB patients with new treatment regimen failure			
4. Children under five years old			
5. People living with HIV/ADIS			
6. Health workers			

7. Not sure who is at high risk to develop MDR-TB			

C7. How can it be possible to prevent MDR-TB?

	1. Yes	2. No	3. I don't know
1. Completing TB treatment properly			
2. Cover mouth and nose when coughing, sneezing, laughing, and talking			
3. Keep the windows and doors open			
4. By using the appropriate treatment regimen/drug for TB			

D. Perceived stigma and discrimination

D1. Please state how much you agree with the following statements:

	Strongly disagree (1)	Disagree (2)	Agree (3)	Strongly agree (4)
1. I feel hurt how others react to knowing I have TB.				
2. I lose friends when I share with them that I have TB.				
3. I feel alone				
4. I keep a distance from others to avoid spreading TB germs				
5. I am afraid to tell those outside my family that I have TB.				
6. I am afraid of going to TB clinics because other people might see me there				
7. I am afraid to tell others that I have TB because they may think that I also have HIV/AIDS.				
8. I feel guilty because my family has the burden of caring for me.				
9. I choose carefully who I tell about having TB.				
10. I feel guilty for getting TB because of my smoking, drinking, or other careless behaviors.				
11. I am worried about having HIV/AIDS.				
12. I am afraid to tell my family that I have TB.				

D2. Please state how much you agree with the following statements:

	Strongly disagree (1)	Disagree (2)	Agree (3)	Strongly agree (4)
1. Some people avoid sharing meals with me because of TB				
2. Some people stay away from me because of TB				

3. Some people avoid inviting me to social activities because of TB				
4. Some people asked me not to come to the work or school because of TB				
5. Some people avoid meeting me because of my TB				
6. Some people asked me to restrict my movement				

D3. Please answer the following questions.

	1. Yes	2. No
1. Have you ever felt you were stigmatized because of your TB status?		
2. Have any of feelings you have about TB inhibited you from seeking and accessing TB services?		
3. Have you experienced stigma in hospitals or clinics that inhibited you from continuing to seek and access TB services?		
4. Have you experienced stigma from your neighbors in your community where you live that inhibited you from seeking and accessing TB services?		
5. Have you experienced stigma at home that inhibited you from seeking and accessing TB services?		
6. Have you experienced stigma at work that inhibited you from seeking and accessing TB services?		

E. Practice of TB Patients

E1. What action was taken first when you experienced cough of at least 2 weeks?

1. Sought care from health care centers
2. Sought care from pharmacies
3. Sought care directly from TB center
4. Did nothing

E2. What is the duration between the date at which you had TB symptom(s) and the date of diagnosis?

[ ] days

E3. What is the duration between the date of TB diagnosis and date of the treatment initiation?

[ ] days

E4. If you think that there was delay in diagnosis or treatment, what are the reasons for the delay?

	1. Yes	2. No
1. Fear of doctors		
2. Financial reasons		
3. Problems with transportation		

4. No family support		
5. Feeling shy for people to know that I have TB		
6. I did not recognize that the symptoms are related to TB symptoms		
7. I was worried about the side effect of TB drugs		
8. I was careless		
9. I was busy with daily life or work		
10. I was not aware of where to go		
11. Health Workers did not diagnose my disease on time		
12. Health Workers ignored my problem		
13. Health Workers did not request investigations on time		
14. Health Workers did not refer me to TB center on time		
15. Health Workers are not friendly		
16. Others, please mention:		

E5. What involvement in TB prevention and control did you make as a TB patient?

	1. Yes	2. No
1. Advised parents to get their infants vaccinated for TB		
2. Advised other TB patients to take their drugs properly		
3. Referred family member to health facility		
4. Others, please mention:		

E6. What practice you use to avoid infecting others?

	1. Yes	2. No
1. I cover my mouth when coughing and sneezing		
2. I dispose sputum correctly and regularly		
3. I stay in a ventilated area and avoid close contact with others		
4. I wear mask		
5. Others, please mention:		

E7. When having TB drugs & get some of its side effects, what will you do?

	1. Yes	2. No
1. Consult with health worker/doctor		
2. Continue the drugs		
3. Stop the drugs and do nothing		
4. Use herbal medicine		

F. Family Support and Heath Workers Support

F1. Please mark (√) under the level of agreement with the following statements.

	Strongly disagree (1)	Disagree (2)	Agree (3)	Strongly agree (4)
1. My family has good relationship with me as before				
2. I am getting better care from family since diagnosis				
3. My family remind me regularly to take the TB drugs				
4. One or more of family members accompany me to receive care				
5. Nobody takes care of me after being diagnosed with TB disease				

F2. Please mark (√) under the level of agreement with the following statements.

	Strongly disagree (1)	Disagree (2)	Agree (3)	Strongly agree (4)
1. Health workers provide me enough time for listening to my problem				
2. During the visiting time, the health workers do motivate me to follow the drug schedule				
3. I am reminded regularly to take the TB drugs by Health worker				
4. Health worker provides me sufficient health education materials				
5. Health workers provide me with comfort sitting place when taking TB drugs				

G. Adherence to medications

G1. Self-reported adherence

	1. Yes	2. No
1. Do you sometimes find it difficult to take your medication?		
2. When you feel better, do you sometimes stop taking your medication?		
3. Sometimes you feel worse after taking your medication, do you stop taking it?		
4. Over the past 4 days, have you missed any of your doses?		

G2. Thinking about the last week when you were taking the medication- How often have you not taken your medication (counting the morning and/or evening as a separate times)?
Please circle the most appropriate answer

Never	1-2 times	3-5 times	6-10 times	Greater than 10 times

H. Patient satisfaction

H1. Please mark (√) under the level of agreement with the following statements.

	Strongly disagree (1)	Disagree (2)	Agree (3)	Strongly agree (4)
1. It was easy to access the clinic for TB treatment				
2. Waiting time in the clinic is reasonable				
3. Doctor listened to me fully, whenever I had any complaint				
4. Health staff were respectful towards me				
5. The amount of time the doctor(s) spent with me during clinic visit was satisfactory				
6. I received good Information about the disease and treatment during first consultation				
7. Privacy during attendance is good				
8. It was easy to make appointments				
9. Clinic is clean				
10. Facilities were available to do examinations in the clinic				
11. Healthcare providers have an excellent professional relationship with me				
12. Healthcare providers spend as much time as I need				
13. Healthcare providers are always available and answer my questions well				
14. Healthcare providers constantly emphasize on the importance of taking my medications as prescribed				
15. Healthcare providers advise me about problems that might occur with my medications (side effects)				

16. Healthcare providers provide me with written information about my drugs and disease				
17. I am happy with the service provided in the center				

H2. What do you think about the TB center services offered?
1. Excellent
2. Very good
3. Good
4. Poor

H3. What do you think about TB health care workers' attitudes toward people who come looking for services?
1. Positive/ Welcoming
2. Negative/ Unwelcoming
3. Neither positive nor negative

H4. What do you think about TB center appearance, in terms of neatness?
1. Excellent
2. Very good
3. Good
4. Poor

I. **Problems faced by patients during COVID-19**

1. Were you diagnosed with TB during the COVID-19 pandemic?

1. Yes 2. No

2. Did get infected with COVID-19?

1. Yes 2. No

3. What would you say about accessing the treatment during the COVID-19 pandemic?

1. Easy 2. Difficult 3. Not possible

4. How was the treatment during the lockdown?
   1. As usual 2. Difficult 3. Not possible
5. Has the health worker been following up with you during the pandemic?
   1. Yes 2. No
6. Was there a focal point person you can contact regarding your treatment during the lockdown?
   1. Yes 2. No
7. Were you able to buy medication from a private pharmacy if not able to visit the hospital?
   1. Yes 2. No
8. Have you or are you facing any stress due to COVID-19 and being ill with TB?
   1. Yes 2. No
9. After the end of the lockdown, have you been following up with the health care provider for health information?
   1. Yes 2. No
10. Did your TB illness made you more alert to become infected with COVID-19?
    1. Yes 2. No
11. Did you face difficulties to take care of families and beloved ones due to TB and isolation measures?
    1. Yes 2. No

12. I avoid crowded places not just due to TB, but also due to COVID-19?
    1. Yes   2. No

J. Clinical Information (*From Medical Records):*

1. Category of TB

☐ New case

☐ Failure

☐ Relapse

☐ Re-treatment (Loss of follow-up)

☐ Other: ___________

2. Treatment

   2.1 Medications:

☐ Isoniazid

☐ Rifampin

☐ Ethambutol

☐ Pyrazinamide

☐ Streptomycin

☐ Other (specify) ____________

   2.2 Phase of treatment

   1. Intensive phase   2. Continuation phase

3. Date of TB diagnosis:
4. Date of Starting TB medication:

Questionnaires.
HIV/AIDS- related KAP questionnaire among patients living with HIV/AIDS

Interview Date (day/mon/yr): ___ ___/____ ___/2021
Interview Time: ___________________________ am \ pm.
Interviewer Name: ________________________________
A. Patients' sociodemographic characteristics

A1. Gender: 1. Male 2. Female	A2. What is your age? [ ] year
A3. What is your marital status?<br><br>1. Single 2. Engaged 3. Married 4. Separated 5. Divorced 6. Widow	
A4. What is the highest education level you have obtained?<br>8. 1. No formal education<br>9. 2. Completed primary school<br>10. 3. Completed middle school<br>11. 4. Completed high school<br>12. 5. Completed 1-2-year post-secondary/vocational<br>13. 6. Completed university/college<br>14. 7. Completed postgraduate degree	
A5. What is your nationality?<br>2. 1. Jordanian 2. Syrian 3. Palestinian 4. Others, __________	
A6. Which of the following best describe your employment status?<br>1. Full-time employed in the workforce (including self-employed)<br>2. Part-time employed in the workforce (including self-employed)<br>3. Unemployed<br>4. Retired or on a pension<br>5. Full-time student<br>6. Part-time student<br>7. Housewife/ Home duties	
A7. What is the best estimate of your monthly household income? [ ] JD<br>*We ask this for statistical purposes. We assure your responses will remain strictly confidential.*	
A8. In the last month, did you have to borrow money to pay any important bills on time, such as electricity, telephone or rent bills?<br>2. 1. Yes 2. No 3. Don't know	
A9. Residency/Governorate:<br>1. Irbid 2. Mafraq 3. Jarash 4. Ajloun 5. Amman 6.Balqa<br>7. Zarqa 8.Madaba 9. Karak 10.Tafilah 11. Ma'an 12. Aqaba	
A10. Do you live with your family? 1. I live alone 2. I live with my family 3. Other: -----------	
A11. Do you now smoke cigarettes? 1. Every day 2. Some days 3. Not at all	
A12. Do you now smoke tobacco in a waterpipe? 1. Every day 2. Some days 3. Not at all	

B. Clinical characteristics

B1. HIV/AIDS diagnosis date? ________________________

B2. Where have you been diagnosed to have HIV/AIDS?
1. Inside Jordan 2. Outside Jordan

B3. In which facility you have been diagnosed?
1. Government hospital
2. Primary healthcare facility
3. Private hospital
4. Public laboratory
5. Private laboratory
6. Others (Specify) ________________________________

B4. Do any of your family members have HIV/AIDS?
1. Yes 2. No 3.I do not know

B5. Do you have any diseases (chronic or acute) other than HIV/AIDS?
1. Yes 2. No 3.I do not know

B6. If your answer to the previous question is (yes), please mention your current diseases.
………

B7. Are you currently taking any medications related to HIV/AIDS?
1. Yes 2. No 3.I do not know

C. Antiretroviral Therapy (ART) status

C1. HIV stage? 1. No symptoms 2. Have symptoms 3. AIDS 4. Unknown
C2. Need ART? 1. Yes 2. No
C3. Using ART? 1. Yes 2. No
C4. Peer-to-peer meeting? 1. Yes 2. No
C5. Have peer support? 1. Yes 2. No
C6. ART duration (year)? ___________

D. Knowledge and awareness about HIV/AIDS

D1. Do you think HIV and AIDS are the same thing?

1. Yes 2. No 3. I do not know

D2. Do you think there is a cure for HIV\AIDS?

1. Yes 2. No 3. I do not know

D3. What is the cause of HIV/AIDS infection?

1. Hereditary 2. Fungal 3. Viral 4. Bacterial 5. I do not know

D4. Do you know the signs and symptoms of HIV/AIDS? 1. Yes 2. No

If your answer to above question is no, please skip the question D5

D5. Which one of the followings are signs and symptoms of HIV/AIDS?

	1. Yes	2. No	3. I don't know
1. Fever that lasts longer than 2 weeks			
2. Lymphadenopathy that lasts longer than 2 weeks			
3. Weight loss			
4. Sore throat that lasts longer than 2 weeks			
5. Rash that lasts longer than 2 weeks			
6. Myalgia/arthralgia that lasts longer than 2 weeks			
7. Diarrhea that lasts longer than 2 weeks			
8. Headache that lasts longer than 2 weeks			
9. Mucocutaneous ulcers			
10. Fatigue that lasts longer than 2 weeks			
11. loss of appetite			

D6. How can HIV/AIDS be transmitted from infected person to another?

	1. Yes	2. No	3. I don't know
1. Unprotected sexual intercourse			
2. Sharing needles between HIV patient and healthy person			
3. HIV can be spread by mosquitoes			
4. A pregnant woman who is HIV infected can transmit the disease to her fetus			
5. A person can get HIV through contact with saliva, tears, sweat, or urine.			
6. HIV can be transmitted through touching the blood of an infected person			

D7. For each of the statements below, please choose correct answer.

	1. True	2. False	3. I don't know
1. People who have been infected with HIV quickly show serious signs of being infected.			
2. There is a vaccine that can stop adults from getting HIV.			
3. A person will NOT get HIV if she or he is taking antibiotics.			
4. A person can get HIV from a toilet seat.			

5. Consistent and correct usage of condom prevents HIV			
6. HIV/AIDS patient may or may not show sign and symptom			

D8. Is there available medication for HIV patients so they can live an ordinary life?
1. Yes
2. No
3. I do not know

D9. Antiretroviral therapy (ART) should be taken throughout the life of the patient
1. Yes
2. No
3. I do not know.

D10. Do you think Antiretroviral therapy helps you to live longer with HIV?
1. Yes
2. No
3. I do not know.

D11. Did you attend any awareness session regarding HIV\AIDS?
1. Yes
2. No
3. I didn’t know about any awareness session.

D12. Do you wish to get more information on HIV?
1. Yes
2. No

D13. What is the main source you refer to if you want to know more about HIV/AIDS? (select one option)

1. Doctor\healthcare providers
2. Scientific references
3. Radio or TV
4. Newspapers or magazines
5. Family, friends, neighbors, or colleagues
6. Internet
7. Others ________

E. Perception and Attitude

E1. Please state how much you agree with the following statements.

	Strongly disagree (1)	Disagree (2)	Agree (3)	Strongly agree (4)
1. A person with HIV/AIDS has no hope				
2. I do not mind if friends or people close to me know that I have AIDS				
3. Having HIV is not the end of one’s life				
4. A person with HIV can get married and have children				
5. HIV/AIDS is a punishment for immoral behavior				
7. It is difficult to prevent HIV/ AIDS transmission				
8. Adherence to treatment is important to improve my quality of life				

F. Stigma and discrimination

F1. How much you agree with the following statements?

	Strongly disagree (1)	Disagree (2)	Agree (3)	Strongly agree (4)
1. People assume I have done something bad to get HIV				
2. Society looks down on people who have HIV				
3. People think that if you have HIV then you got what you deserve				
4. People blame me for having HIV				
5. People assume I slept around because I have HIV				
6. People think that if you have HIV you do not deserve to have children				
7. People think I am a bad person because I have HIV				
8. Medical providers assume people with HIV sleep around				
9. People think you can′t be a good parent if you have HIV				
10. I am concerned if I go to the HIV clinic or AIDS organization, someone I know might see me				
11. I am concerned that people will find out I have HIV by looking at my medical paperwork				
12. I am concerned that if I am sick, people I know will find out about my HIV				
13. Nurses and doctors treat people who have HIV as if they are contagious				
14. Nurses and doctors dislike caring for patients with HIV				
15. I feel abandoned by family members because I have HIV				
16. People avoid me because I have HIV				
17. People I am close to are afraid they will catch HIV from me				
18. I feel like I am an outsider because I have HIV				
19. I feel ashamed to tell other people that I have HIV				
20. I feel alone				
21. I feel guilty because my family has the burden of caring for me.				

F2. How much you agree with the following statements?

	Strongly disagree (1)	Disagree (2)	Agree (3)	Strongly agree (4)
1. I feel that I am treated with less courtesy than other people are because I have AIDS/HIV.				
2. I feel that I am treated with less respect than other people are because I have AIDS/HIV .				
3. People act as if they are afraid of me because you have AIDS/HIV.				

4. I am called names or insulted because I have AIDS/HIV.				
5. I was threatened or harassed because I have AIDS/HIV .				
6. People treat me as less than human now because I have HIV				
7. I have been unfairly fired because I have AIDS/HIV?				

F3. Please answer the following questions.

	3. Yes	4. No
3. Have you ever felt you were stigmatized because of your HIV/AIDS status?		
4. Have any of feelings you have about HIV/AIDS inhibited you from seeking and accessing services?		
5. Have you experienced stigma in hospitals or clinics that inhibited you from continuing to seek and access HIV/AIDS services?		
6. Have you experienced stigma from your neighbors in your community where you live that inhibited you from seeking and accessing HIV/AIDS services?		
7. Have you experienced stigma at home that inhibited you from seeking and accessing HIV/AIDS services?		
8. Have you experienced stigma at work that inhibited you from seeking and accessing HIV/AIDS services?		

G. The social behavior towards HIV

G1. How your family feel about you?

1. feel compassion and desire to help
2. feel compassion but they tend to stay away from me
3. They don’t come close to me
4. Have no feeling

G2. How the community feel about you?

1. The community mostly supports and helps me
2. Most people are friendly but they generally try to avoid me
3. Many people reject me

G3. How is your reaction because you have AIDS/HIV?

	1. Yes	2. No	3. I don't know
1. Fear			
2. Surprise			
3. Shame			
4. Embarrassment			
5. Sadness or hopelessness			

G4. Who knows about your illness?

	1. Yes	2. No	3. I don't know
1. No one			
2. Doctor or health care provider			
3. Some of my family members			
4. All my family members			
5. Close friends			

H. Healthcare seeking behavior

H1. Do you have Medical Insurance? 1. No 2. Yes

H2. Do you pay for HIV medications? 1. No 2. Yes

H3. Where do you usually seek the health care if you are sick?

1. Governmental hospital or clinic
2. Clinic run by an nongovernmental organization
3. Private hospital or clinic
4. Traditional healer
5. Pharmacy

H4. How many times you sought a health care in the last year?

1. Never 2. Once 3. Twice 4. More than two times

H5. What did you do when you got the symptoms of AIDS?

1. Went to health facility
2. Went to pharmacy
3. Went to traditional healer
4. Pursue other self-treatment options (herbs, etc.)
5. Did nothing

H6. When you had symptoms of AIDS, at what point did you seek help from a health facility?

1. As soon as I realize that the symptoms might be related to AIDS
2. When self-treatment does not work
3. When the AIDS symptoms appeared
4. I did not go to a doctor or health facility

H7. When you first had symptoms of AIDS what reasons and difficulties stopped you from going to a health facility? (select all that apply)

1. I didn't have any difficulty and went directly to health facility.
2. Not sure where to go
3. Transport challenges/ distance from health facility
4. Do not trust health workers or health care facilities
5. Cannot leave work
6. Health facility operation hours not convenient
7. Fear to find out that something is wrong.
8. Others, please specify

I. Access to care during COVID-19

I1. Were you diagnosed with HIV/AIDS during the COVID-19 pandemic?
1. Yes 2. No

I2. Did get infected with COVID-19?
1. Yes 2. No

I3. What would you say about accessing HIV treatment during the COVID-19 pandemic?
1. Easy 2. Difficult 3. Not possible

I4. How was the treatment received during the lockdown?
2. As usual 2. Difficult 3. Not possible

I5. Has the health worker been following up with you during the pandemic?
2. Yes 2. No

I6. Was there a focal point person you can contact regarding your treatment during the lockdown?
2. Yes 2. No

I7. Were you able to buy medication from a private pharmacy if not able to visit the hospital?
2. Yes 2. No

I8. Have you or are you facing any stress due to COVID-19 and being ill with HIV/AIDS?
2. Yes 2. No

I9. After the end of the lockdown, have you been following up with the health care provider for health information?
2. Yes 2. No

I10. Did your HIV/AIDS illness made you more alert to become infected with COVID-19?
2. Yes 2. No

I11. Did you face difficulties to take care of families and beloved ones due to HIV/AIDS and isolation measures?
2. Yes 2. No

I12. I avoid crowded places due to COVID-19?
2. Yes 2. No

J. Practices

J1. Do you have a sexual partner? 1. Yes 2. No

J2. Have you had sex in the last three months? 1. Yes 2. No

J3. How regularly do you use condom during sexual intercourse?

1. Always 2. Sometimes 3. Never

J4. Have you had used shared/unsterile syringes/needles in the last three months?

1. Yes
2. No
3. I do not remember

J5. Have you had used alcohol or drugs in the last three months?

4. Yes
5. No
6. I do not remember

<u>J6. Treatment-related Practices</u>

J6a. In the last week, that is the last 7 days, how many days did you miss taking your ART?

1. 0 days
2. 1-6 days
3. 7 days

J6b. If missed taking your ART, what are the reasons?

J7c. Frequency of visits to the HIV center

1. Every 3 months
2. Every 4-6 months
3. Every 7-12 months
4. Once a year

J8d. CD4 test/ viral load tests frequency

1. Every 0-6 months
2. Every 7-12 months
3. Once or less a year

K. Patient satisfaction

K1. Please mark (√) under the level of agreement with the following statements.

	Strongly disagree (1)	Disagree (2)	Agree (3)	Strongly agree (4)
18. It was easy to access the clinic for HIV/AIDS treatment				
19. Waiting time in the clinic is reasonable				
20. Doctor listened to me fully, whenever I had any complaint				
21. Health staff were respectful towards me				
22. The amount of time the doctor(s) spent with me during clinic visit was satisfactory				
23. I received good Information about the disease and treatment during first consultation				
24. Privacy during attendance is good				
25. It was easy to make appointments				
26. Clinic is clean				
27. Facilities were available to do examinations in the clinic				

28. Healthcare providers have an excellent professional relationship with me				
29. Healthcare providers spend as much time as I need				
30. Healthcare providers are always available and answer my questions well				
31. Healthcare providers constantly emphasize on the importance of taking my medications as prescribed				
32. Healthcare providers advise me about problems that might occur with my medications (side effects)				
33. Healthcare providers provide me with written information about my drugs and disease				
34. I am happy with the service provided in the center				

K2. What do you think about the center services offered?

5. Excellent
6. Very good
7. Good
8. Poor

K3. What do you think about the health care workers' attitudes toward people who come looking for services in this center?

4. Positive/ Welcoming
5. Negative/ Unwelcoming
6. Neither positive nor negative

K4. What do you think about this center appearance, in terms of neatness?

5. Excellent
6. Very good
7. Good
8. Poor

L. Adherence to ART

L1. Please answer the following statements regarding adherence to your medications:

	1. Yes	2. No
5. Do you sometimes find it difficult to take your medication?		
6. When you feel better, do you sometimes stop taking your medication?		
7. Sometimes you feel worse after taking your medication, do you stop taking it?		
8. Over the past 4 days, have you missed any of your ART doses?		

L2. Thinking about the last week- How often have you not taken your medication (counting the morning and/or evening as a separate times)?

Please circle the most appropriate answer

Never	1-2 times	3-5 times	6-10 times	Greater than 10 times

© 2023 Teshima A. et al.
